# Views of Aging During the Corona Pandemic

**DOI:** 10.1093/geroni/igab046.2261

**Published:** 2021-12-17

**Authors:** Jennifer Bellingtier, Anna Kornadt

## Abstract

In the Covid-19 pandemic, media stories and government reports have emphasized the heightened risk of being “old” and placed a spotlight on the way we think and talk about older adults and aging. In this symposium we investigate how the pandemic and the public discourse about older adults has shaped views of aging in different countries. Bellingtier et al. report on German children’s views of older adults before and during the pandemic. Children placed greater distance between themselves and older adults both before and after the pandemic, suggesting early ageism in children that predates the pandemic. Levy et al. provide experimental evidence that media stereotypes about aging and Covid-19 influence the mental health of older American adults, both in positive and negative. Schwartz and Ayalon found that greater perceptions of age-based discrimination in the healthcare system by Israeli adults 50+ were significantly related to greater Covid-19 worries. Greater worry can motivate older adults to take precautions, but be detrimental if it becomes too high. In line with this finding, Tingvold et al. found in a study with older adults from Luxembourg that more Covid-19 worry predicted feeling older four months later, but only for those in worse health. Finally, Terracciano examined longitudinal change in subjective age and found that American adults reported feeling younger after the emergence of Covid-19 than before, suggesting that perceptions of aging partly reflect a coping process to counter the negativity in the media.

